# Comparison of Fludarabine/Treosulfan Conditioning with Melphalan-Based Sequential Conditioning in Patients with MDS and Increased Blasts Undergoing Upfront Transplantation

**DOI:** 10.3390/cancers18203303

**Published:** 2026-10-14

**Authors:** Judith Metzdorf, Artur Schneider, Jacob Pyka, Annemarie Mohring, Jennifer Kaivers, Tim Lohmann, Hans Christian Reinhardt, Nicolaus Kröger, Christina Rautenberg, Thomas Schroeder

**Affiliations:** Department of Hematology and Stem Cell Transplantation, West German Cancer Center Essen, University Hospital Essen, Hufelandstr. 55, 45147 Essen, Germany

**Keywords:** myelodysplastic syndromes, upfront transplantation, fludarabine/treosulfan, sequential conditioning, melphalan

## Abstract

Background: Upfront allogeneic stem cell transplantation (allo-SCT) without pretransplant cytoreduction is an established concept in patients with myelodysplastic syndromes with increased blasts (MDS-IB), but the optimal conditioning strategy remains to be defined. While fludarabine and treosulfan (Flu/Treo) has become an established regimen for older patients based on its favorable ratio between toxicity and efficacy, its impact when used in the context of upfront transplant is less well described. Methods: Here, we retrospectively investigated Flu/Treo as the backbone for conditioning in 35 patients with MDS-IB (median blast count 13%) receiving upfront transplantation. Results: Outcomes in this high-risk cohort (75% IPSS-M high or very high) were promising, with an estimated 2-year overall (OS), relapse-free survival (RFS) and non-relapse mortality (NRM) of 76%, 56% and 13%, respectively. Relapse was the major cause of treatment failure, reflected by a relapse incidence (CIR) of 31%. We identified the TP53 mutation status as the main driver of relapse (2-year RFS TP53 mutated 17% vs. 67%, *p* = 0.01; 2-year CIR 54% vs. 24%, *p* = 0.08) and an adverse prognostic factor, while the pretransplant blast count did not affect the post-transplant outcome. Intensification with the addition of melphalan (Mel, *n* = 18) to a sequential conditioning approach did not improve the outcome (Mel vs. Flu/Treo 2-year OS 62% vs. 87%, RFS 50% vs. 63%, CIR 37% vs. 24%, NRM 14% vs. 12%, all not significant). Conclusions: This pilot series shows that upfront transplantation using Flu/Treo conditioning in high-risk MDS patients facilitates promising post-transplant outcomes and may serve as a backbone to further improve these results through innovative peri-transplant interventions.

## Simple Summary

Upfront allo-SCT without pretransplant cytoreduction is an established concept in MDS patients with increased blasts (MDS-IB), but the optimal conditioning strategy remains to be defined. Therefore, we retrospectively analyzed Flu/Treo conditioning and Mel-based sequential conditioning in 35 patients with MDS-IB in the upfront setting. In our study, conditioning with Flu/Treo is effective and safe for these patients but the intensification by the addition of melphalan does not improve outcomes. Further, the outcomes seem to be independent of the pretransplant blast count.

## 1. Introduction

Myelodysplastic syndromes (MDSs) are heterogeneous hematopoietic stem cell disorders, and allogeneic stem cell transplantation (allo-SCT) is the only curative treatment option up to now. In case of an increased bone marrow blast count (MDS-IB), there is controversy among experts regarding the need for pretransplant cytoreduction using either hypomethylating agents (HMAs) or intensive chemotherapy (ICT) [1,2]. Pretransplant debulking strategies aim to reduce the disease burden in order to reduce the risk of relapse after transplant, but may cause morbidity or even mortality. This may negatively impact the post-transplant outcome or even preclude individual patients undergoing a transplant. In the absence of randomized prospective studies, a direct so-called upfront allo-SCT without pretransplant cytoreduction has emerged as an established alternative for these patients, and retrospective comparisons suggest at least non-inferiority [3,4]. In this context, so-called sequential conditioning, which combines an intensive debulking chemotherapy and the actual conditioning regimen within one step, is often employed [5,6]. Initially, this approach was pioneered in patients with relapsed/refractory disease acute myeloid leukemia (AML), but it is also used in patients with MDS-IB in the upfront transplant setting. While the so-called FLAMSA protocol probably represents the most common sequential regimen, melphalan (Mel)-based sequential conditioning is a valuable alternative, facilitating comparable outcomes in patients with AML [7,8,9]. In patients with MDS, Mel-based sequential conditioning is less well described so far. Furthermore, reduced-toxicity conditioning with fludarabine and treosulfan (Flu/Treo) has become the standard for patients with MDS, and recent results suggest that its success might be independent of the disease burden prior to transplant in terms of bone marrow (BM) blasts [10,11,12].

Therefore, this retrospective single-center study aimed first to investigate the efficacy and safety of an upfront transplantation using a Flu/Treo backbone for conditioning of patients with MDS-IB. In a second step, we addressed the impact of intensification by the addition of Mel to this backbone as a sequential conditioning approach.

## 2. Methods

### 2.1. Study Design

For this analysis, we included all consecutive MDS patients who had received their first allo-SCT at our institution between 2018 and 2024. As a prerequisite for inclusion, all patients had to have a BM blast count of 5–19% at the time of transplantation, reflecting the optional indication for pretransplant cytoreduction [1]. Still, no patient had received any pretransplant, remission-inducing cytoreductive therapy prior to transplant, but all had received an upfront transplantation. Conditioning consisted either of fludarabine/treosulfan (Flu/Treo) conditioning or melphalan-based sequential conditioning. Flu/Treo conditioning consisted of fludarabine (30 mg/m^2^; d −6 to −2) and treosulfan (10–12 g/m^2^; day −4 to −2). Sequential conditioning included melphalan (100–140 mg/m^2^) on day −11, followed by fludarabine (30 mg/m^2^; d −6 to −2) and treosulfan (10–12 g/m^2^; day −4 to −2). The decision between the two conditioning strategies was based on a change in the center philosophy. The range of the melphalan dosage was customized based on age and comorbidities, with no significant differences between the two treatment groups. The dosage range of treosulfan used here relates to the drug development of treosulfan, where daily dosages of 10 to 14 g/m^2^ were tested, and as a result of a randomized phase-3 trial, were finally established and approved. In vivo T-cell depletion was performed with either anti-T-lymphocyte globulin (ATG; Grafalon^®^, Neovii, Rapperswil-Jona, Switzerland) in the case of fully matched donors or post-transplant cyclophosphamide (PTCy) in the case of mismatched donors. Immunosuppression consisted of a calcineurin inhibitor and mycophenolate mofetil. All patients gave written informed consent for scientific use of their data. This study was performed in accordance with the Declaration of Helsinki and approved by the local ethics committee of the University Duisburg/Essen (approval number: 22-10708-BO). Data lock for this analysis was 1 November 2025.

### 2.2. Definitions

MDS subtypes were categorized according to the WHO guidelines 2022, and for risk stratification, the Molecular International Prognostic Scoring System (IPSS-M) was used [13,14]. The Hematopoietic Cell Transplantation-specific Comorbidity Index (HCT-CI) and graft-versus-host disease (GvHD) were specified as described previously [15,16,17]. Neutrophil engraftment was defined as the first of three consecutive days, with a measure of >0.5 × 10^9^/L, while platelet engraftment was defined as the first of three consecutive days, with a measure of >20 × 10^9^/L.

### 2.3. Statistical Analyses

Categorial variables are displayed as frequencies, and comparisons were performed using cross-tabulation and Fisher’s Exact test, while continuous variables were summarized by the median (range) and analyzed for differences between subgroups by employing the Mann–Whitney U test. Overall survival (OS) was calculated from the time point of allo-SCT until death by any cause or the date of follow-up. Relapse-free survival (RFS) was defined as the time from SCT until relapse or death, whichever occurred first, and surviving patients without relapse were censored at the date of the last follow-up. Probabilities of survival were estimated by the Kaplan–Meier method, and log-rank tests were applied for univariable comparisons. The cumulative incidence of relapse (CIR) and non-relapse mortality (NRM) were considered as competing risks and calculated using a cumulative incidence estimator with Gray’s test for univariable comparisons. A *p*-value of <0.05 was considered significant. Statistical analysis was performed using Excel (Microsoft Corp, Redmond, WA, USA) including the add-on XLSTAT, version 2024.4.2 (Lumivero, Denver, CO, USA), and R, version 4.31 (R Core Team, Vienna, Austria).

## 3. Results

### 3.1. Patient-, Disease- and Transplant Characteristics

In total, 35 patients, who had received an upfront transplantation between 2018 and 2024 and had ≥5% BM blasts at the time of transplantation (median BM blast count 13%, range 5 to 19%) were included. Detailed patient characteristics are given in Table 1 for the entire cohort as well as for the 18 patients (51%) who had received Mel-based conditioning, and the 17 patients (49%) with Flu/Treo conditioning separately. The median age was 63 years (range, 30–75 years), 54% had MDS-IB2, 66% had an abnormal karyotype, including 29% with a complex karyotype, and 75% had an IPSS-M high or very high, thereby reflecting the high disease risk of this cohort. Baseline patient and disease characteristics including the BM blast count at the time of transplant were equally distributed between the two treatment groups. The only factor differing between the two groups was a significantly higher frequency of an abnormal karyotype in patients receiving Mel-based conditioning (Table 1). As depicted in Table 2, most transplant-related factors were well balanced between the two groups. Nevertheless, more patients in the Flu/Treo group received a graft from matched related donors, and consequently, in vivo T-cell depletion was used less frequently in this treatment group.

### 3.2. Posttransplant Outcome

After a median follow-up of 37 months (range, 5–79), the estimated 2-year OS, RFS, CIR and NRM probabilities of the entire cohort were 76% [95% CI: 62–94%], 56% [95% CI: 41–77%], 31% [95% CI: 16–48%] and 13% [95% CI: 4–28%], respectively (Figure 1 and Table 3). When comparing the outcomes after transplantation according to the two conditioning regimens, we did not observe significant differences regarding OS (2-year OS: Mel 62% [95% CI: 40–97%] vs. Flu/Treo 87% [95% CI: 72–100%]; *p* = 0.13), RFS (2-year RFS: Mel 50% [95% CI: 30–84%] vs. Flu/Treo 63% [95% CI: 44–92%]; *p* = 0.35), CIR (2-year CIR: Mel 37% [95% CI: 14–60%] vs. Flu/Treo 24% [95% CI: 7–47%]; *p* = 0.37) and NRM (2-year NRM: Mel 14% [95% CI: 2–38%] vs. Flu/Treo 12% [95% CI: 2–33%]; *p* = 0.99). In a next step, we compared the two conditioning strategies with regard to GvHD incidence and severity as well as hematopoietic recovery (Table 3). There were no significant differences in terms of frequency and severity of aGvHD. Still, a higher proportion of patients receiving Flu/Treo developed chronic GvHD (cGvHD incidence: Mel 33% vs. Flu/Treo 71%; *p* = 0.04), which is probably related to the lower number receiving in vivo T-cell depletion in the Flu/Treo-treated patients. While time to neutrophil recovery (≥0.5/nl) was comparable between the two regimens, platelet engraftment (≥20/nl) was significantly slower in patients receiving Mel-based conditioning (median time to 20/nl platelets: Mel 20 days vs. Flu/Treo 13 days; *p* = 0.0002).

Besides the conditioning strategy, we tested several patient-, disease- and transplant-related factors with regard to their impact on post-transplant outcomes. In univariable analyses, the presence of a *TP53* mutation was associated with a lower RFS and a higher relapse incidence (Table 4). The finding of a higher NRM and a lower RFS in female patients is probably related to biological heterogeneity and the limited sample size (Table 4).

## 4. Discussion

The optimal conditioning for patients with MDS with increased blasts receiving potentially curative allo-SCT remains to be defined. This is particularly the case for patients undergoing so-called upfront transplantation without pretransplant cytoreduction. Our retrospective analysis on 35 consecutive MDS patients with increased blasts shows that upfront transplantation following Flu/Treo-based conditioning results in promising outcomes after transplantation for this elderly high-risk cohort. Furthermore, augmentation of the conditioning with the addition of melphalan as a sequential conditioning approach does not seem to improve the outcome compared to conditioning with Flu/Treo alone.

The value of “debulking” cytoreductive therapy either with HMA or chemotherapy in patients with MDS-IB is not properly defined. In the absence of prospective, randomized trials on this relevant clinical question, results from retrospective analyses indicate that upfront transplantation is at least comparable, and that debulking approaches, especially if not successful in reducing the disease burden prior to transplant, may even be disadvantageous [3,18,19,20,21,22,23]. In these analyses investigating upfront transplantation, various conditioning regimens were used and facilitated 5-year OS rates ranging between 41% and 66% [3,21,23]. In this context, our 2-year OS rate of 76% combined with an NRM rate of 13% in the entire cohort appears promising and supports the notion from the above-mentioned reports that upfront transplantation is a valuable concept in patients with increased blasts.

We uniformly employed a Flu/Treo backbone for conditioning in all patients investigated in our analysis here. The combination of Flu/Treo has become a standard for elderly, comorbid patients with MDS and AML as consequence of a randomized phase-3 trial, which demonstrated superior event-free and overall survival as well as a lower NRM after Flu/Treo in comparison to busulfan conditioning [10,24]. In this prospective trial, around half of the MDS patients had received pretransplant therapy, and 45% had <5% BM blasts as well as very low to intermediate disease according to IPSS-R [10,12,24]. In contrast, our analysis included only patients with ≥5% BM blasts, with 75% belonging to the IPSS-M high or very high risk category, who all received Flu/Treo as part of an upfront transplantation concept. Interestingly, a BM blast count at the time of transplantation below or above 10% did not impact outcomes in our cohort, which supports findings from a previous analysis from our center [11] and a subgroup analysis from the pivotal phase-3 trial [25]. Overall, the promising results in terms of the 2-year OS and low NRM rate observed in our entire cohort underline the favorable ratio between efficacy and tolerability of Flu/Treo as a toxicity-reduced myeloablative conditioning regimen for patients with MDS in the context of upfront transplantation [26].

Previous analyses suggest that upfront transplantation may not ultimately reduce the risk of relapse, but in the case of relapse patients, who had received upfront transplantation, they seem to better respond to hypomethylating agent-based salvage therapy [27,28,29]. Indeed, the relapse incidence following upfront transplantation with Flu/Treo conditioning was 31% at 2 years in our cohort of high-risk MDS patients. This is in accordance with previous reports on the use of upfront transplantation using various conditioning strategies [3,30] and indicates that, with the use of Flu/Treo, relapse remains the major cause of treatment failure. One option aiming to reduce the risk of relapse in upfront transplantation could be the use of sequential conditioning, which combines intensive AML-like induction therapy and conditioning within one step. In the context of upfront transplantation in MDS patients, the so-called FLAMSA approach has already been employed, with relapse rates ranging from 22% to 46% [3,23,30]. In about half of the patients in our analysis, the Flu/Treo backbone was augmented by melphalan to sequential conditioning, which appeared to be equivalent in terms of outcome compared to other sequential conditioning regimes in the relapsed/refractory AML setting [31]. Still, we failed to improve the outcome with the addition of Mel, since the OS and relapse incidence were comparable to those of the patients who received Flu/Treo only. This finding, that augmentation to sequential conditioning does not improve outcomes in patients with MDS, is in line with results from a larger retrospective EBMT registry as well as a single-center analysis [30,32]. Vice versa, it emphasizes the general efficacy of Flu/Treo conditioning, even though it seems not to be able to overcome the disease-specific, non-modifiable risk of adverse genetics such as TP53. On the one hand, this calls for the incorporation of novel approaches such as venetoclax into the conditioning [33]. On the other hand, post-transplant interventions such as maintenance or pre-emptive therapy may help to improve the outcomes in these high-risk patients. Regarding toxicity, we observed a significant delay in platelet engraftment in the Mel group, pointing towards potentially slower engraftment kinetics in sequential conditioning. This may be associated with clinical and resource utilization implications, such as a bleeding risk, higher transfusion requirements and prolonged hospitalizations. On the other hand, there was a higher rate of cGvHD in the Flu/Treo group. Since significantly more patients in the Flu/Treo group received grafts from MRD, but fewer patients underwent in vivo T-cell depletion, the finding of a higher cGvHD rate is probably related to these baseline immunotherapeutic and donor differences, rather than a biological protective effect of Mel-based sequential conditioning.

We have to acknowledge some relevant limitations owing to the limited number of patients and the retrospective character of our analysis without prospective randomization between Mel-based sequential conditioning and Flu/Treo only. Along with this, the absence of statistical significance in survival and relapse outcomes between the cohorts could represent a Type II error (false negative), indicating the need for confirmation in larger patient groups. Still, we tried to follow a stringent policy change, implementing melphalan in all patients transplanted from 2021 independent of their disease properties to avoid a major selection bias. This is reflected by the mostly balanced disease characteristics between the two treatment groups. Nevertheless, there were more patients with an abnormal karyotype in the Mel group, which may reflect the adverse disease biology potentially associated with worse post-transplant outcomes. Still, in terms of high-risk genetic features such as a complex karyotype, TP53 mutation and IPSS-M risk category reflecting cytogenetics and mutations, there was no difference between the two groups. In terms of transplant-related factors, significantly more patients in the Flu/Treo group received grafts from MRD, while fewer patients underwent in vivo T-cell depletion. Thus, the higher rate of cGvHD in these patients is mainly related to these baseline immunotherapeutic and donor differences, rather than a biological protective effect of Mel-based sequential conditioning.

## 5. Conclusions

In summary, our pilot series on upfront transplantation using Flu/Treo in high-risk MDS patients shows that this conditioning approach is safe and effective and facilitates promising post-transplant outcomes independent of the BM blast count. The augmentation by melphalan seems not to improve the outcome, although this needs to be further investigated and validated in prospective, randomized trials with larger patient numbers. Finally, Flu/Treo conditioning may serve as a backbone, which may be further improved by the incorporation of novel pretransplant cytoreductive approaches, novel compounds as part of the conditioning, and post-transplant interventions.

## Figures and Tables

**Figure 1:**
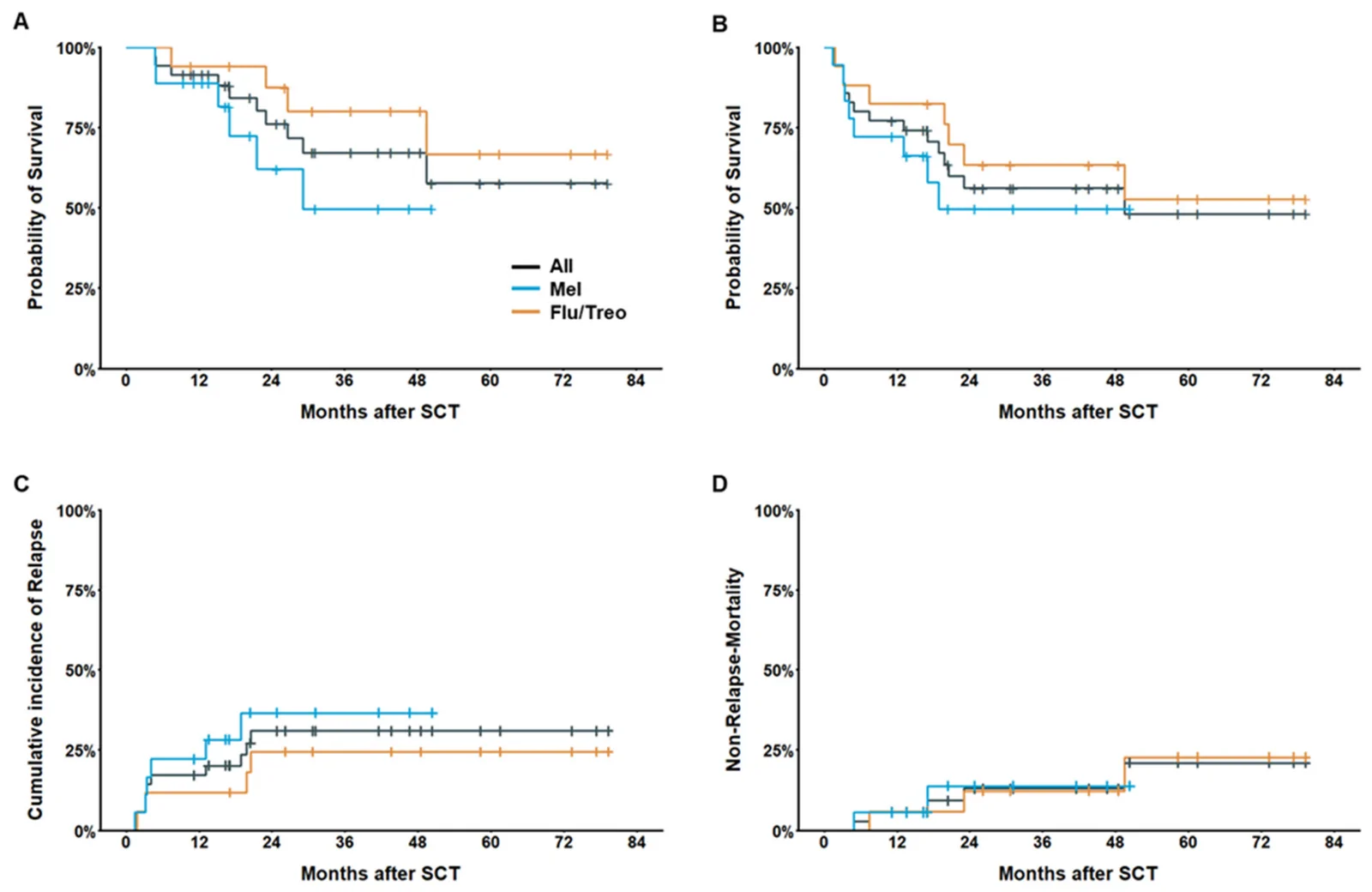
Posttransplant outcomes of MDS-IB patients. OS (**A**), RFS (**B**), CIR (**C**) and NRM (**D**) are presented for the entire cohort (black), the Mel group (blue) and the Flu/Treo group (orange).

**Table 1:** Disease characteristics.

Characteristic	No. (%)			
	All pts (*n* = 35)	Mel (*n* = 18)	Flu/Treo (*n* = 17)	*p*
Age at ID, years (median, range)	63 (30–75)	63 (46–70)	60 (30–75)	0.53
Sex of patient female male	15 (43) 20 (57)	9 (50) 9 (50)	6 (35) 11 (65)	0.50
WHO 2022 diagnosis MDS-biTP53 MDS-LB MDS-IB1 MDS-IB2 CMML-1 CMML-2	3 (9) 2 (6) 7 (20) 19 (54) 1 (3) 3 (9)	1 (6) 1 (6) 3 (17) 11 (61) 0 (0) 2 (11)	2 (12) 1 (6) 4 (24) 8 (47) 1 (6) 1 (6)	0.87
Therapy-related MN yes no	3 (9) 32 (91)	1 (6) 17 (94)	2 (12) 15 (88)	0.60
IPSS-M (*n* = 31 ^#^) low moderate low moderate high high very high	1 (3) 1 (3) 6 (19) 7 (23) 16 (52)	1 (6) 0 (0) 3 (19) 4 (25) 8 (50)	0 (0) 1 (7) 3 (20) 3 (20) 8 (53)	1.00
Karyotype normal abnormal complex	12 (34) 23 (66) 10 (29)	3 (17) 15 (83) 4 (22)	9 (53) 8 (47) 6 (35)	0.02 ^ 0.47 ^^
TP53 mutation yes no	8 (23) 27 (77)	2 (11) 16 (89)	6 (35) 11 (65)	0.12
HCT-CI, *n* missing = 1 low intermediate high	11 (32) 6 (18) 17 (50)	6 (33) 5 (28) 7 (39)	5 (31) 1 (6) 10 (63)	0.23
BM blast count at ID (median, range) <10% ≥10%	11 (3–19) 16 (46) 19 (54)	15 (3–19) 5 (28) 13 (72)	10 (5–15) 11 (65) 6 (35)	0.05
BM blast count at Tx (median, range) <10% ≥10%	13 (5–19) 13 (37) 22 (63)	14 (5–19) 7 (39) 11 (61)	10 (5–19) 6 (35) 11 (65)	0.98

BM, bone marrow; CMML, chronic myelomonocytic leukemia; Flu/Treo; fludarabine/treosulfan; HCT-CI, hematopoietic cell transplantation—comorbidity index; ID, initial diagnosis; IPSS-M, molecular international prognostic scoring system; LB, low blasts; IB, increased blasts; MDS, myelodysplastic syndrome; Mel, melphalan; MN, myeloid neoplasm; pts, patients; Tx, transplantation. ^#^ without CMML patients. ^ normal vs. abnormal. ^^ complex vs. non-complex.

**Table 2:** Transplant characteristics.

Characteristic	No. (%)			
	All pts (*n* = 35)	Mel (*n* = 18)	Flu/Treo (*n* = 17)	*p*
Age at Tx, years (median, range)	63 (31–75)	65 (46–70)	60 (31–75)	0.73
Time between ID and Tx, months (median, range)	4 (2–57)	4 (2–20)	4 (3–57)	0.92
T-cell depletion no yes ATG PTCY	4 (11) 31 (89) 24 (69) 7 (20)	0 (0) 18 (100) 13 (72) 5 (28)	4 (24) 13 (76) 11 (65) 2 (12)	0.03
Stem cell source BM PB	3 (9) 32 (91)	2 (11) 16 (89)	1 (6) 16 (94)	1.00
CD34 total ×10^6^/kg bw (median, range)	7 (1–12)	7 (1–12)	7 (1–11)	0.55
Donor type MRD MUD MMUD	6 (17) 24 (69) 5 (14)	0 (0) 14 (78) 4 (22)	6 (35) 10 (59) 1 (6)	0.008 ^ 0.34 ^^
Sex donor female male	12 (34) 23 (66)	5 (28) 13 (72)	7 (41) 10 (59)	0.49
CMV status d/r pos/pos pos/neg neg/neg neg/pos	17 (49) 2 (6) 13 (37) 3 (9)	9 (50) 1 (6) 7 (39) 1 (6)	8 (47) 1 (6) 6 (35) 2 (12)	1.00 °

ATG, anti-T-lymphocyte globulin; BM, bone marrow; bw, body weight; CMV, cytomegalovirus; d, donor; Flu/Treo; fludarabine/treosulfan; ID, initial diagnosis; Mel, melphalan; MMUD, mismatched unrelated donor; MRD, matched related donor; MUD, matched unrelated donor; neg, negative; PB, peripheral blood; pos, positive; PTCY, post-transplantation phosphhamide; pts, patients; r, recipient; Tx, transplantation. ^ related vs. unrelated. ^^ matched vs. mismatched. ° recipient CMV pos vs. neg.

**Table 3:** Transplant outcomes.

Characteristic	No. (%)			
	All pts (*n* = 35)	Mel (*n* = 18)	Flu/Treo (*n* = 17)	*p*
2 yr OS [95% CI]	76 [62; 94]	62 [40; 97]	87 [72; 100]	0.13
2 yr RFS [95% CI]	56 [41; 77]	50 [30; 84]	63 [44; 92]	0.35
2 yr CIR [95% CI]	31 [16; 48]	37 [14; 60]	24 [7; 47]	0.37
2 yr NRM [95% CI]	13 [4; 28]	14 [2; 38]	12 [2; 33]	0.99
ANC recovery after allo-SCT days (median, range) yes no	17 (10–26) 34 (97) 1 (3)	17 (10–26) 18 (100) 0 (0)	18 (11–20) 16 (94) 1 (6)	0.90 0.49
PLT recovery after allo-SCT days (median, range) yes no	19 (11–40) 34 (97) 1 (3)	20 (14–40) 18 (100) 0 (0)	13 (11–33) 16 (94) 1 (6)	0.0002 0.49
Acute GvHD no yes ≥Grade III	14 (40) 21 (60) 4 (11)	10 (56) 8 (44) 2 (10)	4 (24) 13 (76) 2 (12)	0.09
Chronic GvHD no yes severe	17 (49) 18 (51) 4 (11)	12 (67) 6 (33) 1 (6)	5 (29) 12 (71) 3 (18)	0.04

Allo-SCT, allogeneic stem cell transplantation; ANC, absolute neutrophile count; CI, confidence interval; CIR, cumulative incidence of relapse; Flu/Treo; fludarabine/treosulfan; GvHD, graft-versus-host disease; Mel, melphalan; NRM, non-relapse mortality; RFS, relapse-free survival; OS, overall survival; PLT, platelet; pts, patients; SCT, stem cell transplantation; yr, year.

**Table 4:** Univariable analysis.

Variable	2 yr OS *p* (%)		2 yr RFS *p* (%)		2 yr CIR *p* (%)		2 yr NRM *p* (%)	
Median age ≤63 years (*n* = 18) ˃63 years (*n* = 17)	81 69	0.08	66 44	0.16	24 40	0.34	6 20	0.14
Sex of patient female (*n* = 15) male (*n* = 20)	52 89	0.20	33 72	0.03	34 28	0.46	33 0	0.05
IPSS-M low/moderate high (*n* = 8) high/very high (*n* = 23)	71 85	0.98	71 57	0.36	14 29	0.42	14 14	0.76
HCT-CI low/intermediate (*n* = 17) high/very high (*n* = 17)	77 74	0.81	58 49	0.70	30 36	0.88	12 16	0.63
Karyotype normal (*n* = 12) abnormal (*n* = 23)	90 67	0.50	57 55	0.66	45 22	0.37	0 21	0.06
TP53 yes (*n* = 8) no (*n* = 27)	56 82	0.08	17 67	0.01	54 24	0.08	29 8	0.42
Blast count at Tx <10% (*n* = 13) ≥10% (*n* = 22)	58 85	0.30	42 63	0.21	25 32	0.96	30 4	0.06
Donor type related (*n* = 6) unrelated (*n* = 29)	83 74	0.47	50 58	0.92	34 30	0.94	17 13	0.96
Sex of donor female (*n* = 13) male (*n* = 23)	69 79	0.59	33 68	0.18	27 39	0.64	28 5	0.20
CMV-status recipient negative (*n* = 15) positive (*n* = 20)	71 80	0.22	55 57	0.70	30 31	0.80	16 11	0.90

CMV, cytomegalovirus; CIR, cumulative incidence of relapse; Flu/Treo; fludarabine/treosulfan; HCT-CI, hematopoietic cell transplantation—comorbidity index; IPSS-M, molecular international prognostic scoring system; Mel, melphalan; NRM, non-relapse mortality; RFS, relapse-free survival; OS, overall survival; Tx, transplantation; yr, year.

## Data Availability

The supporting data of this study are available upon reasonable request from the corresponding author.
